# The Physiological and Biochemical Outcomes Associated with a Reflexology Treatment: A Systematic Review

**DOI:** 10.1155/2014/502123

**Published:** 2014-05-05

**Authors:** J. E. M. McCullough, S. D. Liddle, M. Sinclair, C. Close, C. M. Hughes

**Affiliations:** Institute of Nursing and Health Research, University of Ulster, Jordanstown Campus, Shore Road, Newtownabbey, County Antrim BT37 0QB, UK

## Abstract

*Background*. Reflexology is one of the top forms of complementary and alternative medicine in the UK and is used for healthcare by a diverse range of people. However, it is offered by few healthcare providers as little scientific evidence is available explaining how it works or any health benefits it may confer. The aim of this review was to assess the current evidence available from reflexology randomised controlled trials (RCTs) that have investigated changes in physiological or biochemical outcomes. *Methods*. Guidelines from the Cochrane Handbook of Systematic Reviews of Interventions were followed: the following databases were searched from inception to December 2013: AMED, CAM Quest, CINAHL Plus, Cochrane Central Register of Controlled Trials, Embase, Medline Ovid, Proquest, and Pubmed. Risk of bias was assessed independently by two members of the review team and overall strength of the evidence was assessed using the Grading of Recommendations, Assessment, Development, and Evaluation guidelines. *Results*. Seventeen eligible RCTs met all inclusion criteria. A total of 34 objective outcome measures were analysed. Although twelve studies showed significant changes within the reflexology group, only three studies investigating blood pressure, cardiac index, and salivary amylase resulted in significant between group changes in favour of reflexology. The overall quality of the studies was low.

## 1. Introduction 

Reflexology is considered to be a form of complementary and alternative medicine (CAM). CAM refers to treatments used either as an adjunct to, or instead of conventional medical care. The House of Lords Select Committee for Science and Technology [1] has placed reflexology in group two, categorised as therapies used mostly to complement conventional medicine. Its popularity has increased in recent years as the public seek more holistic ways to maintain good health and well-being [2]. In fact CAM is increasingly being considered as a safe and effective way of reducing the causes and impacts of pain and disease.

Reflexology is one of the top six forms of CAM used in the UK [3] and, according to a survey by McDonough et al. [4], it is the second most popular form of CAM used in Northern Ireland. In 2007 a national survey in the USA reported that 38% of adults and 12% of children were using some form of CAM [5] and in the same year a Norwegian survey indicated that 5.6% of the population had used reflexology in the preceding twelve months [6]. In support, a national survey carried out in Denmark in 2005 showed that 21% of the population had used reflexology at some point in their lives and 6% had used it within the previous year [7]. In the UK there are thought to be over 40,000 complementary therapists and the sector is expected to grow by over 30% from *£*213 million in 2009 to *£*282 million by 2014 [8].

While the general population is spending large sums of money on CAM, health care professionals are reluctant to promote any benefits for such treatments. Reflexology has come under much criticism based predominantly on the dearth of high quality evidence supporting a clear scientific mechanism of action for the treatment. Most of the research carried out in this area has investigated the psychological outcomes from reflexology focusing on qualitative outcomes. Researchers have repeatedly shown that reflexology has a positive effect on quality of life, stress, anxiety, and pain [9–12]. However, few studies have attempted to correlate these results with any quantitative physiological or biochemical outcomes.

To date six literature reviews of reflexology have been carried out [13–18] and three further reviews on reflexology for cancer care [19], pain and fatigue [2], and pain management [11] are also available. None of these have focused specifically on the quantitative aspect of the results available, although the overwhelming opinion from the authors is that there is not enough high quality RCTs to produce significant scientific data for recommending reflexology as an evidence-based treatment option.

The exact mechanism of action of reflexology has yet to be confirmed; however, various theories have been proposed and Tiran and Chummun [20] have detailed many of the current theories in their paper. One of the earliest is the haemodynamic theory which suggests that reflexology stimulation enhances blood flow to the corresponding organ or body part [21]. The findings of an investigation using colour Doppler sonography [22] showed a significant effect on blood flow to the kidney during reflexology and an investigation by Jones et al. [3] displayed some evidence to support this theory. Recent research has also indicated that changes in the dermal layer structures and luminosity of the skin at specific reflex points on the feet may give rise to the changes felt by therapists during a treatment [23]. The “nerve impulse theory” proposes that stimulation applied to specific reflex points on the feet enhance nervous connection to the corresponding body parts [24]. This is currently the most promising theory and suggests that the benefits of reflexology may be brought about by modulation of the autonomic nervous system (ANS). The ANS regulates body systems that are under unconscious control such as breathing, heart rate, and blood pressure. These parameters are sensitive to stressors and fluctuate according to the physical or psychological changes experienced by an individual, via vagal modulation which controls calming and restful changes and regular functioning or sympathetic modulation, responsible for controlling arousal and the “fight or flight” response. ANS modulation has also been supported by Hughes et al. [25] and Sliz et al. [26]. A commonly held belief, often cited by reflexologists and suggested by Poole et al. [27] and Tiran [28], states that reflexology may work by stimulating the release of endorphins and in this way may help to reduce pain and increase feelings of wellbeing and relaxation.

## 2. The Review

### 2.1. Aim

This systematic review aimed to assess the quality of evidence from RCTs that have tested changes in physiological or biochemical outcome parameters as a result of reflexology.

### 2.2. Design

**Figure 1:**
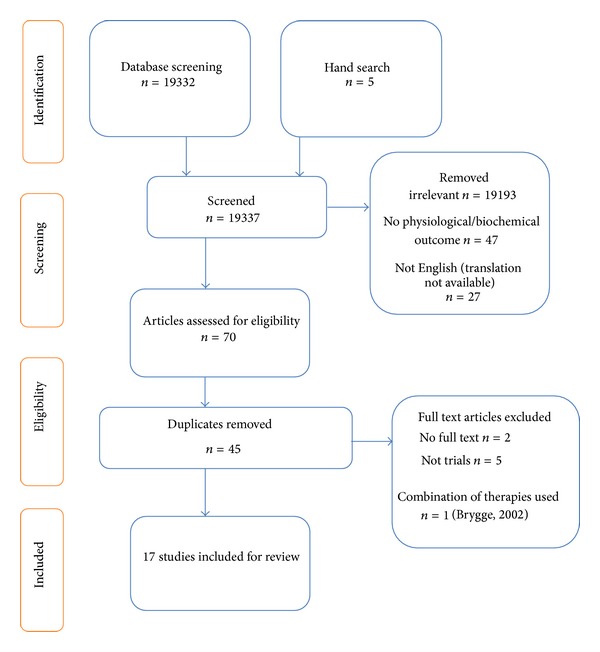
Systematic literature search PRISMA flow diagram.

The Cochrane guidelines for conducting systematic reviews were followed throughout this review. An inclusion and exclusion protocol was devised to determine which studies were included for evaluation, and the risk of bias (ROB) of each eligible RCT was assessed independently by two members of the review team. The PRISMA guidelines [43] were used (Figure 1) and the methodological quality of each trial was determined by carrying out an ROB assessment following guidelines in the Cochrane Handbook for Systematic Reviews [44]. The strength of the evidence for the RCTs was assessed according to the constructs of the GRADE (Grading of Recommendations, Assessment, Development, and Evaluation) tool [29] adapted to suit this review (Table 1).

### 2.3. Search Methods

The following databases were searched from their inception to December 2013: AMED, CAM Quest, CINAHL Plus, Cochrane Central Register of Controlled Trials, Embase, Medline Ovid, Proquest, and Pubmed. Search terms used in various combinations were “reflexology, blood, urine, saliva, plasma, electrolyte, hormone, neurotransmitter, neuroregulator, immune response, lymph, lymphatic system, respiratory function, respiratory function tests, blood pressure, heart rate, foetal heart rate, fetal heart rate and endorphin”. Hand searches of relevant journals and reference lists along with citation tracking were undertaken to ensure comprehensive coverage of all relevant literature. The references of all articles were hand searched. Identified publications were read either as abstracts or full texts.

### 2.4. Search Outcome

A total of 19337 articles were retrieved via the initial database and hand searches. Twenty-three studies remained after the exclusion of irrelevant studies (19193), those with no biochemical or physiological outcome measure (47), those articles not available in English (27), duplicates (45), and those where the full text was not available (2). Another five studies were excluded as they were not RCTs, and one further RCT was excluded as it used a combination of reflexology plus back massage for the intervention group [45]. Therefore, 17 papers were extracted for systematic review and critical appraisal. The risk of bias is detailed in Table 2.

### 2.5. Data Abstraction and Synthesis

All results were screened by two independent reviewers and differences in opinion were resolved through discussion to reach a consensus. Exclusions were applied using the criteria in Table 3. In addition, when insufficient information was available from the paper, the review team contacted the study authors.

**Figure 2:**
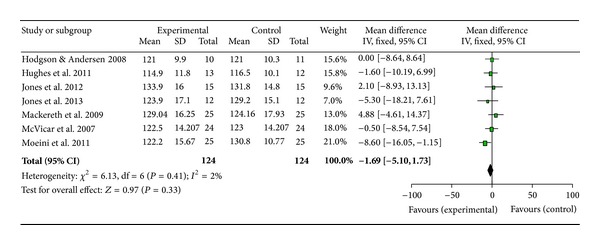
Meta-analysis and forest plot of systolic blood pressure.

**Figure 3:**
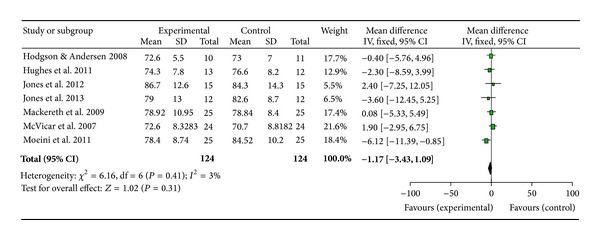
Meta-analysis and forest plot of diastolic blood pressure.

**Figure 4:**
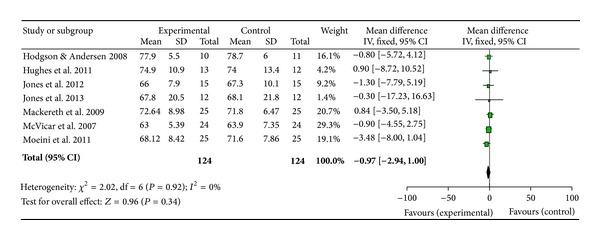
Meta-analysis and forest plot of heart rate.

Owing to the consistent use of blood pressure (BP) and heart rate as outcome measures in the studies collated, a meta-analysis was conducted using Review Manager 5.2 to gain further insight into the effects of reflexology across a wide range of populations (Figures 2, 3, and 4).

## 3. Results

Twelve randomised controlled trials and five feasibility or pilot randomised controlled trials, involving a total of 697 participants, were reviewed (Table 4). The trial participants ranged from healthy volunteers to those suffering from a wide range of musculoskeletal, neurological, and systemic conditions including people with breast cancer, coronary artery bypass graft (CABG), chronic heart failure (CHF), nursing home residents with dementia and cancer, chronic obstructive pulmonary disease (COPD), multiple sclerosis (MS), pregnant women, and women failing to ovulate.

In total, 34 physiological or biochemical outcome measures were analysed in the 17 included studies. Significant within reflexology group changes were recorded for 11 outcome measures. These were blood pressure in five studies, heart rate in three studies, cortisol in two studies, salivary amylase, lymphocyte production, heart rate variability (HRV), R-R interval, pulse pressure, cardiac output, cardiac index (CI), and blood oxygen level dependant (BOLD) response, in one study each (Table 4). However, only four outcome measures showed significant changes between the reflexology and control group: salivary amylase [38], systolic and diastolic blood pressure [34], and CI [3]. Ruiz-Padial et al. [32] demonstrated significant changes in blood pressure as a factor of time, treatment number, and intervention. Eight of the studies compared reflexology to an active CAM control instead of sham treatment and eleven used a control measure which involved touching the participants feet. In one study the same reflexology treatment was compared in two different participant groups [33].

## 4. GRADE Analysis

The strength of the evidence presented by the 17 included RCTs was assessed using the GRADE criteria which resulted in the quality of evidence being rated as very low (Table 5). However, assessment must be viewed with caution. The GRADE start score was four, the maximum available, which indicated that all the studies included had an RCT design which is considered the gold standard [44]. Study limitations surrounding blinding were considered serious (−2). However, blinding of participants, therapists, and outcome assessors in reflexology trials is extremely challenging as sham treatment to the feet that mimics reflexology is difficult to achieve without some active stimulation of reflexology points. Neither can any therapist be truly blinded as they must have prior knowledge of protocols and whether a true or sham treatment is to be employed. Table 2 shows the trend towards high ROB for blinding of participants and, in particular, therapists. The GRADE score was further marked down for serious inconsistencies between studies and indirectness of results (−1). However, due to the nature of this review, studies were not comparable given that different outcome measures and populations were assessed. All included studies had small sample sizes which resulted in a further downgrade due to imprecision.

## 5. Physiological Outcome Measures

### 5.1. Cardiac Parameters

The physiological parameter most commonly investigated within reflexology studies was BP, with 13 studies including this as an outcome measure. BP responded in a positive manner to reflexology in half of the studies: Mollart [10], Mc Vicar et al. [40], Mackereth et al. [37], Hughes et al. [25], Lu et al. [33], and Moeini et al. [34]. The work by Moeini et al. [34] was the only trial to show a significant difference between the treatment and control groups.

Contrary to all of the other studies in this review Ruiz-Padial et al. [32] found that BP increased following reflexology, foot massage, and sitting quietly in a room. However, average BP results at baseline were different for each of the three study groups and the results presented were as a function of time, treatment number, and treatment interaction which made them difficult to interpret.

Changes in heart rate (HR) were investigated in nine studies and positive results were demonstrated by Mc Vicar et al. [40], Wilkinson et al. [41], and Mackereth et al. [37]; however, none were statistically significant.

Jones et al. [3] investigated a wide range of cardiac parameters with cardiac index (CI) only having a significant change between the active and control groups. A within reflexology group change in cardiac output (CO), the volume of blood being pumped by the heart per minute, was significant compared with baseline.

### 5.2. Functional Magnetic Resonance Imaging (fMRI)

fMRI involves the use of MRI to detect visible changes that occur in the brain as a result of some external stimuli. Sliz et al. [26] investigated the effects of foot treatments on brain activity using fMRI and evaluated the findings using the blood oxygen level demand (BOLD) response which relates to changes in blood flow to the brain during activation. The results showed positive BOLD responses for reflexology, Swedish massage and the rest only control group, but not in response to massage with a wooden roller massager.

## 6. Biochemical Outcome Measures

A total of seven studies investigated a biochemical response to reflexology. These studies tended to focus on stress hormones and were correlated with qualitative data on stress and anxiety levels.

Significant within group decreases in cortisol were found by Mackereth et al. [37] and Hodgson and Lafferty [31] but not by Mc Vicar et al. [40]. However, no between group differences were found in these studies. No changes were observed for melatonin [40] or progesterone [36]. However, Hodgson and Andersen [38] found significant reductions in salivary amylase within the reflexology group. Green et al. [35] found a significant increase in CD25+ cells, involved in cancer cell death and tumour growth inhibition, within the reflexology and control massage groups.

## 7. Meta-Analysis

A meta-analysis was performed on seven papers investigating BP and HR [3, 25, 30, 34, 37, 38, 40]. The study by Wilkinson et al. [41] was not included as only one participant completed all parts of the study, Mollart [10] presented no data and was therefore, excluded, and Gunnarsdottir and Jonsdottir [39] and Ruiz-Padial et al. [32] were also excluded as no results means or standard deviations were available to the research team. Mackereth et al. [37] provided results for both arms of their crossover trial; however, since results did not return to baseline during the washout period, only data from the experiment which delivered the reflexology treatment first was included in the meta-analysis. The forest plots show estimated effect for systolic BP as 1.69 (Figure 2), diastolic BP as 1.17 (Figure 3), and heart rate as 0.97 (Figure 4) all in favour of reflexology.

## 8. Discussion

The focus of this paper was to review the evidence available from RCTs investigating any quantitative physiological or biochemical outcome measure as a result of reflexology as there has been minimal evaluation to date. Seventeen studies were included for review from a total of 19337 articles identified. A notable limitation, however, is the exclusion of studies not available in English, owing to a lack of availability of translation of these papers, along with these not having sufficient data to perform a meta-analysis; this would have undoubtedly further informed the review outcomes.

Only three RCTs in this review represented a significant change between the reflexology intervention and the control group [3, 34, 38]. These studies showed few similarities; however, Hodgson and Andersen [38] and Moeini et al. [34] employed a control measure that involved no touch, which may have reduced the incidence of the placebo effect, or patient therapist interaction in the control group that could otherwise have reduced the difference between groups. Jones et al. [3] investigated a wide range of cardiac parameters in healthy volunteers with only CI showing a significant between groups difference. However, using the same methodology in heart failure patients resulted in no changes. This may suggest that the positive effects achieved in healthy individuals in one treatment may require multiple treatments in participants with health conditions or that the treatment may not be transferable between the different populations. Interestingly Gunnarsdottir and Jonsdottir [39] used a similar participant group and study design to Moeini et al. [34] but no significant between group changes were determined (a significant reduction in systolic BP within the control group was reported). These results show that the effects of reflexology are not repeatable across all groups.

Within group significant changes were observed for eight outcome measures across a range of ten studies, and a further four studies resulted in no significant changes for either the intervention or control group (Table 4). Importantly, significant within group changes that do not achieve enough strength to lead to between group changes may be due to several external factors and therefore must be viewed in light of this. Reasons for within group changes may be due to a regression to the mean, an unknown difference between groups at baseline, the normal effect of time or participants becoming accustomed to the treatment, therapist, or setting, or a change in health status of the participants. Interestingly, the studies by Mc Vicar et al. [40] and Mackereth et al. [37] both employed a crossover design and both showed a within group change for HR and BP which may have influenced baseline data and results in the reflexology group.

Reflexology is an area which has seen much growth within the private sector; however, little work has been carried out to determine a possible “mode of action” or how it may be best incorporated into mainstream medical care from a measurable, quantitative perspective informed by high quality evidence. This review highlights that while reflexology has seen minimal investigation over the past 20 years, the hypothesised mechanism of action has been focused on the modulation of the ANS. However, the scope of the research has been very broad, therefore making it difficult to draw any firm conclusions due to the lack of consistency in participant groups, outcome measures, methodologies, and evaluation techniques.

The studies considered in this review were undertaken in a range of countries demonstrating that reflexology is considered to be worthy of investigation and also a socially acceptable form of treatment globally. Nine studies were carried out in the UK, six of which were performed in National Health Service (NHS) hospitals. In total, ten trials were completed in hospitals, four in universities, and three in nursing homes in the following countries: UK (nine studies), USA (two studies), and one each in the following countries: Australia, Canada, Iceland, Iran, Spain, and Taiwan. Although half of the studies carried out in the UK had a low ROB, neither country nor location had any discernible effect on the ROB.

To date relatively small studies have been carried out, with the mean number of participants per study being 41; in a well-designed, 3-armed trial this would result in less than 14 participants per group. Only five of the named studies carried out power calculations prior to recruitment. Mackereth et al. [37] determined a total requirement of 46 participants and a total of 53 patients were recruited resulting in statistically significant results in BP and cortisol levels. Hughes et al. [25] included ten participants per group for their feasibility study. The authors went on to complete a post hoc analysis which indicated that 180 participants would be required for a fully powered study to achieve 90% power. Mollart [10] estimated a required sample size of 120, which was later reduced to 60, determined by a smaller initial population than first expected. Holt et al. [36] stated that a post hoc analysis found that at least 600 participants would be necessary to determine any connection between reflexology and ovulation. Jones et al. [30] based their calculation of twelve participants on previous research using the same protocol and a healthy population. This would indicate that, in general, the lack of solid statistically significant data is the result of small study sizes (imprecision); therefore larger trials are required to adequately test the effects of reflexology.

Studies involving reflexology have investigated a wide range of outcome parameters using a range of measurement methods. Treatments have been applied to various groups of different ethnicity and gender and with different illnesses, using a wide range of experimental designs, measurements, and analyses. This has led to a very low quality evidence as stated earlier, demonstrating that the RCTs that have been carried out in this area and the results should be treated with caution. It has also demonstrated that overall, with respect to reflexology, low quality studies have been carried out and those where the ROB was low were small and yielded few statistically significant results. However, it is important to note that high ROB is largely due to the lack of participant and therapist blinding. Importantly ROB analysis has demonstrated that studies involving reflexology, and indeed any touch therapy, are complex and difficult to design in order to maintain adequate blinding status for the participants, therapists, and assessors alike. Ultimately, if statistically significant between groups differences from low ROB studies are analysed, this would give rise to only one study [3] demonstrating the effectiveness of reflexology, which is a very weak body of evidence for 17 years' worth of research activity.

In the six studies with a low ROB for participant blinding (Table 2) this was achieved in each case by using a comparison treatment that was an alternative form of reflexology. All of the studies reviewed showed a high ROB for clinician blinding. This highlights the difficulty of using an adequate control treatment that renders the participant blind to the treatment received and the therapist impartial to the allocation of treatment. These difficulties in participant blinding persisted even where an alternative foot massage treatment was used as the control measure [26, 31]. Interestingly participants in the intervention group in the study carried out by Frankel [42] were reported as not having had reflexology before. They were given either true or sham reflexology, although no data on blinding index was reported. More recently, Hughes et al. [25] excluded volunteers who had previous experience of reflexology. However, 66.7% of the control group receiving the foot hold control measure reported that they were in the control group. This may illustrate that the general population is becoming more aware of reflexology and what it involves, thus allowing them to reliably assert whether they are receiving true reflexology or not. Therefore, future RCTs involving CAM will require more complex designs with greater emphasis on comparing and contrasting different treatments.

Indeed it is a limitation of any study involving reflexology to employ a suitable sham treatment that will allow the participants to remain blind to the intervention but have no therapeutic effect, as even gentle pressure on the feet may give rise to stimulation of a reflex point of interest. Eleven of the RCTs used a control treatment which also involved touching the feet. The results of those studies, which used sham reflexology, foot hold, cream application, nonprofessional massage, or Swedish massage, showed fewer differences between groups. Conversely studies where the control measure involved no touch showed more statistically significant changes between groups and more positive changes in outcome measures suggesting that touch plays an integral part in the response to reflexology.

The number of treatments participants received ranged from one 4.5-minute treatment to seven separate one-hour treatments. The duration and frequency of treatments did not have any effect on the results. Likewise, the type of reflexology performed did not appear to have any effect.

A major difficulty for researchers and therapists alike is the various different types of reflexology used. This current review demonstrated this with six different types of reflexology employed, including the Bayly method, Ingham method, Father Josef method, a gentle method developed at Anglia Ruskin University, UK, by Mc Vicar et al. [40], reflexotherapy, and hand reflexology. The most commonly cited protocol in seven studies was the Ingham method or a derivation of this; however, only five studies stated any precise reflexology routine. As with other therapies, research and experience have resulted in generic reflexology evolving into specific forms particular to a school of thought, or the practices and findings of individual therapists. Also each reflexology map may have subtle differences leading to difficulties in pinpointing specific reflexes. The points which seem to most commonly come into question are the heart, solar plexus, and pituitary gland. This lack of precision and agreement may result in a perceived lack of validity of reflexology among medical professionals and other health care providers. Hodgson and Andersen [38] stated that patients received hand or foot reflexology; however, no further data on numbers was given which may have offered valuable insight into the effects of two different types of reflexology within the same population. In accordance with most texts, reflexology is most usually carried out right foot first; interestingly, in their study, Moeini et al. [34] treated the left foot first. The absence of written details about the routines used during research studies renders them impossible to repeat by other researchers keen to investigate the method or critique it in the light of changing trends in reflexology application. Future studies should aim to provide detailed methods of treatments carried out. Furthermore, a lack of consistent terminology exists within the field of reflexology and CAM modalities in general [38], adding a further level of confusion to therapists, researchers, and medical professionals alike.

A further potential confounder for these studies may lie in the number of therapists providing reflexology treatments during trials. During the research for this review one study boasted 32 therapists taking part; however, the authors did not cite this as a possible limitation of the study. A single reflexologist delivering treatments may generally use the same technique and employ the same treatment type and schedule per client; however, in only seven of the 17 RCTs in this review was it stated that a single therapist carried out all the treatments. Small fluctuations in treatment type or even the mannerisms of the therapist may have an important effect on treatment outcomes.

Only two studies stated whether music was played during treatments [3, 40]. Relaxing music is commonly played during the delivery of reflexology. Music therapy is an allied health profession. A Cochrane Heart Group review [46] states that listening to music may have a beneficial effect on blood pressure, heart rate, respiratory rate, anxiety, and pain in persons with coronary heart disease (CHD). Therefore, as it may have a positive effect on treatment outcomes, it is important to standardise the use of music during trials investigating complementary therapies.

While this review focused on the physiological and biochemical outcomes recorded for reflexology interventions, the literature available clearly shows that for all of the articles evaluated, whether a significant change was identified or not, reflexology had a positive effect on the health and well-being, quality of life, stress, anxiety, and pain levels of the participants involved. Hodgson and Lafferty [31] indicated that cortisol reduction correlated with a reduction in pain and stress and Hodgson and Andersen [38] found salivary amylase to be significantly reduced between the reflexology and control group, corresponding to a significant reduction in pain. This is in keeping with the ethos that reflexology is a therapy that aims to promote harmony of mind, body, and soul. Reflexology reduces stress and anxiety levels [3, 32, 36–38, 40] and increases feelings of well-being and quality of life [35]. The paper by Holt et al. [36] revealed promising evidence for reflexology. The participants in the control arm received sham reflexology and the study had a low ROB for participant blinding. In this study the Hospital Anxiety and Depression (HAD) scale results also showed a significant between groups reduction in favour of “true” reflexology suggesting some added value with “true” reflexology. Swedish massage to the feet has been shown to reduce stress [26] by activation of the sACC region of the brain and this may go some way to explain how reflexology helps to reduce stress levels, given that massage is an integral part of a complete reflexology treatment. No papers were found that investigated any other biochemical related to stress or pain.

This review is the first to carry out a meta-analysis of papers investigating BP and HR. These were the most commonly analysed outcome measures, likely due to the ease and noninvasive nature of recording these parameters. While the forest plots appear to show positive benefits in favour of reflexology, cautious interpretation of the results is needed. The clinical heterogeneity of the studies, the mix of healthy and non-healthy populations, the variation in control interventions and the low number of participants (124) would result in a low quality evidence. Furthermore the confidence intervals for all results cross zero and, therefore, the results must not be viewed as significant at this stage until more data becomes available and further analyses can be carried out.

Overall, the review indicates that only three studies resulted in significant between group differences [3, 34, 38] suggesting that reflexology can result in a significant reduction in cardiac index (CI) in healthy volunteers, salivary amylase in elderly dementia patients, and BP in patients prepared for coronary artery bypass graft (CABG). These results are supported by the meta-analyses; however it does not provide any firm conclusions. CI is a measure of how well the heart is functioning to pump blood around the body, suggesting a link between reflexology stimulation and cardiac blood flow and circulation. These parameters are also functions of stress and regulated by the ANS. Overall, had the studies been larger a more significant change in quantitative stress outcome measures may have been demonstrated correlating with the overwhelming evidence for a reduction in qualitative indicators. Therefore, reflexology should be promoted for any medical condition where stress is contraindicated.

While no firm scientific evidence for the effective and efficacious use or “mode of action” of reflexology has been established, it is nonetheless currently being used in healthcare settings around the world including hospices, nursing homes, and maternity departments. In many countries reflexology is associated with the beauty industry or traditional unorthodox medicine. However, attitudes to CAM therapies are shifting towards their use as secondary medical healthcare and integrating them into mainstream medicine. Ten studies in this review were carried out in hospitals suggesting a more positive attitude of health professionals towards the use of CAM therapies as potential adjuncts to mainstream medical healthcare. Thus, this review has implications globally for all health professionals seeking innovative and novel methods for patient care.

There were no serious adverse effects related to any of the treatments for any of the studies. Ruiz-Padial et al. [32] stated that the participants in the reflexology intervention experienced more discomfort that those receiving a foot massage; however, both groups rated the pleasantness of the treatments they received as high. Commonly reflexology may cause some discomfort if the particular body part or organ corresponding to the reflex point being worked is out of balance, or in a state of disease [47, 48]. However, care should be taken by the therapist to avoid causing any unnecessary pain or discomfort during treatments as this would have a negative effect on the outcomes of the treatment. Reflexology is deemed suitable for almost all individuals. It has been shown to reduce stress in the elderly with life limiting disease [31] and therefore may be an effective adjunct to palliative care for patients, their carers, and their families. It also reduces BP, which the National Institute of Health and Care Excellence (NICE) estimated cost the NHS one billion pounds in 2006 in drug charges alone [49]. It is considered to be a safe, noninvasive, and inexpensive form of healthcare accessible by the vast majority of the population including children, the very elderly, terminally ill patients, and pregnant women [48]. Therefore, in the current economic climate investing in research into reflexology may prove dividends for health and the provision of healthcare globally.

Overall reflexology has a positive effect on health, reducing physiological and psychological stress. However, it is as yet unclear how this specialised foot massage exerts its action and whether physiological stress parameters are reduced due to a reduction in psychological stress or vice versa.

## 9. Conclusions

This systematic literature review is the first, to our combined knowledge, to specifically analyse the existing data available from RCTs investigating the physiological and biochemical changes associated with reflexology, and it has demonstrated that a range of positive effects can be attributed to the treatment, specifically a reduction in stress parameters. This will inform health care professionals of the evidence base for known benefits and will enhance evidence based decision making at clinical level. It is important to note that, in all of the studies included in this review, where psychological parameters were assessed, a significant improvement in health and well-being was determined and this factor alone had a positive effect on disease outcomes, prognosis, and rehabilitation. None of the studies in this review investigated any long-term effects through follow-up with participants. Therefore, this is an aspect of CAM study design that must be addressed in the future.

It is still unclear from this review precisely how reflexology impacts physiological and biochemical parameters. It illustrates the need for further research into the use, efficacy, and mode of action of reflexology with well designed, high quality RCTs, if indeed RCTs are a suitable mode of investigation. Also, this review highlights the need for further research into the measurable physiological and biochemical effects of reflexology in order to address the concerns of healthcare professionals and thus allowing all patients to benefit from any positive outcomes afforded by this inexpensive, noninvasive, and nonpharmacological therapy. It is more than likely, however, that a number of factors are at work, both of a physiological and psychological nature and that reflexology is what it attests to be, a treatment that seeks to enhance and harmonise the mind, body, and spirit.

## Figures and Tables

**Table 1:** GRADE evaluation guidelines [29].

Study limitations	The quality of the evidence was downgraded if serious study limitations existed such as a lack of allocation concealment, lack of blinding, large loss to follow-up, or randomized trials stopped early for benefit or the selective reporting of outcomes.

Inconsistency	The quality of the evidence was downgraded if there was inconsistency in the results, for example, if studies showed varying or different effects of the same intervention.

Indirectness	The quality of the evidence was downgraded if there was a level of indirectness in the studies, for example, if interventions had not been compared directly to one another or if the studies investigated a restricted version of the main review question in terms of population, intervention, or outcomes.

Imprecision	The quality of the evidence was downgraded if the studies were imprecise in any respect, for example, if they included few participants and few events and thus had wide confidence intervals.

Publication bias	The quality of the evidence was downgraded if some element of reporting bias was evident, for example, authors failed to report all the outcomes they set out to or perhaps only reported the positive findings of their study.

**Table 2:** Risk of bias (ROB) analysis.

Study	Type of study	Adequate sequence generation	Allocation concealment	Adequate blinding-participant	Adequate blinding- clinician	Adequate blinding-outcome assessor	Incomplete outcome data assessment	Selective reporting bias	Other bias	Risk of bias
Jones et al., 2013 [30]	RCT	Low	Low	Low	High	Low	Low	Low	Low	Low
Hodgson and Lafferty, 2012 [31]	Pilot	Unclear	Unclear	High	High	Low	Low	Low	Low	Unclear
Jones et al., 2012 [3]	RCT	Low	Low	Low	High	Low	Unclear	Low	Low	Low
Ruiz-Padial et al., 2012 [32]	RCT	Unclear	Unclear	Low	High	Unclear	Low	Low	Low	Unclear
Sliz et al., 2012 [26]	RCT	Low	Low	High	High	Unclear	Low	Low	Low	Low
Hughes et al., 2011 [25]	Pilot	Low	Low	Low	High	Low	Low	Low	Low	Low
Lu et al., 2011 [33]	RCT	Unclear	Unclear	Low	Unclear	Unclear	Low	Unclear	Unclear	Unclear
Moeini et al., 2011 [34]	RCT	Low	High	High	High	High	Unclear	Low	Low	Unclear
Green et al., 2010 [35]	RCT	Low	Low	High	High	Unclear	Low	Low	Unclear	Low
Holt et al., 2009 [36]	RCT	Low	Low	Low	High	Low	Low	Low	Low	Low
Mackereth et al., 2009 [37]	RCT	Low	Low	High	High	Unclear	High	Low	Unclear	Unclear
Hodgson and Andersen, 2008 [38]	RCT	High	High	High	High	Low	Unclear	High	Unclear	High
Gunnarsdottir and Jonsdottir, 2007 [39]	Pilot	Unclear	Unclear	Unclear	High	High	Unclear	Low	Low	Unclear
Mc Vicar et al.,2007 [40]	Pilot	Unclear	Unclear	High	High	High	High	High	Low	High
Wilkinson et al., 2006 [41]	RCT	Unclear	Unclear	High	High	Unclear	High	High	High	High
Mollart, 2003 [10]		Unclear	High	High	High	High	High	High	Unclear	High
Frankel, 1997 [42]	Pilot	Unclear	Low	Unclear	High	Unclear	Low	Low	Low	Unclear

**Table 3:** Systematic literature search inclusion and exclusion criteria.

Inclusion criteria	Exclusion criteria
Foot reflexology treatment only	Self-treatment only
A quantitative biochemical outcome measure	Qualitative outcome measure only
A quantitative physiological outcome measure	Full text not available
Randomised controlled trials	Full article not available in English
Pilot studies	Duplicate

**Table 4:** Table of study characteristics.

Study	Participants	Intervention	Comparison	Dropouts	Outcome measure	Adverse effects	Results	Comments
Jones et al., 2013 [30] (Scotland, UK) Double-blind RCT	12 patients with stable chronic heart failure	4.5 mins reflexology to heart reflex area (active heart point) (Ingham Method)	4.5 mins reflexology on gross heel area	No dropouts occurred	Beat-to-beat cardiovascular parameters HR, BP, stroke index (SI), cardiac output (CO), cardiac index (CI), total peripheral resistance (TPR), baroreceptor up/down events (BarUpEv)/barDwEv), and heart rate variability (HRV)	None	No sig. difference for any outcome for either group	The authors state that participants medication may have masked any potential benefit

Hodgson and Lafferty, 2012 [31] (USA) Pilot crossover RCT with one week's washout	18 older cancer survivors in nursing homes	4 × 20 mins reflexology (Ingham Method)	4 × 20 mins Swedish massage to feet	No dropouts occurred	Salivary cortisol	None reported	Sig. change for both groups, no sig. difference between group	No details regarding whether cortisol levels returned to baseline during the washout period were given

Jones et al., 2012 [3] (Scotland, UK) Double-blind RCT	16 healthy volunteers	4.5 mins reflexology to heart reflex area (active heart point) (Ingham method)	4.5 mins reflexology on gross heel area	1 due to data collection issue	Beat-to-beat cardiovascular parameters HR, BP, stroke index (SI), cardiac output (CO), cardiac index (CI), total peripheral resistance (TPR), baroreceptor up/down events (BarUpEv)/(BarDwEv), heart rate variability (HRV)	None reported	Sig. decrease in CI for intervention group, sig. change in CO from baseline for intervention group, no other sig. results, sig. between group difference for CI	Suggests a link between reflexology stimulation to the heart reflex area and cardiac blood flow and circulation

Ruiz-Padial et al., 2012 [32] (Spain) RCT	41 healthy volunteers	3 × 40 mins reflexology (Ingham Method)	3 × 40 mins (1) Non-professional foot massage (2) Seated in a darkened room	None reported	BP, BRS, HRV, Inter-beat interval (IBI)	Some pain reported for reflexology group	Increases in interbeat interval, HRV and BRS in all groups. Sig. increase in BP in reflexology group as a function of time	The authors state that the increase in BP in the reflexology group suggest a “co-activation of the two branches of the ANS,” namely, the sympathetic and parasympathetic pathways

Sliz et al., 2012 [26] (Canada) RCT	40 healthy volunteers used a mental stress test to increase stress levels	1 × 8.5 mins reflexology to right foot only	1 × 8.5 mins (1) Swedish foot massage (2) Foot massage with a wooden object to mimic Swedish massage (3) Rest	None reported	fMRI, blood oxygen level dependent (BOLD) response (indicated blood flow to areas of activation)	None reported	Positive BOLD response in ACC and PCC brain region for reflexology, Swedish massage and control, no sig. difference between groups	The ACC and PCC regions of the brain are thought to be linked to emotional response, learning, and memory and are also involved in major depressive disorders (Dervets et al., 2008)

Hughes et al., 2011 [25] (Northern Ireland, UK) Feasibility RCT	25 healthy volunteers using a mental stress test to increase stress levels	1 × 20 mins reflexology	1 × 20 mins relaxation and foot hold	No dropouts occurred	BP, HR	None reported	Sig. reduction in SBP for intervention and control groups, sig. reduction in DBP in intervention group, no sig. change in HR, no sig. difference between groups	

Lu et al., 2011 [33] (Taiwan) RCT	37 participants	1 × 60 mins reflexology (Father Josef Method) CAD patients	1 × 60 mins reflexology (Father Josef Method) healthy patients	None reported	ECG, BP, HRV, PP, RRI	None reported	Sig. reduction in BP and PP for both groups, sig increase in RRI in reflexology group. Sig. change in HRV in both groups, no sig. difference between groups	HRV benefits lasted longer for CAD patients (60 mins) compared with controls (30 mins)

Moeini et al., 2011 [34] (Iran) RCT	50 CABG patients	1 × 30 mins reflexotherapy pre-surgery	Usual care	None reported	BP, HR, respiratory rate	None reported	Sig reduction in SBP and DBP in reflexology group, nonsignificant reduction in HR and respiration rate for reflexology, sig. between groups difference in SBP and DBP	

Green et al., 2010 [35] (UK) RCT	183 Early stage breast cancer (6 weeks post-surgery)	8 × treatment (weekly session duration unknown)	(1) Self-initiated support (2) 8 treatments at weekly intervals of SIS with head massage	Full data sets were obtained for 87 participants, intention to treat was carried out	Blood lymphocytes (CD profiles) cytokine production (Th1, Th2), prolactin, cortisol, growth hormone	None reported	Sig. increase in CD25 + cells in reflex and massage group compared with baseline. Sig. increase in CD25 + cells between massage and SIS, no other sig. results, no sig. difference between groups	Results for only 47.5% of the participants were reported due to a loss of blood sample in the analysis process

Holt et al., 2009 [36] (UK) RCT	49 Women with anovulation	7 × 60 mins reflexology	Sham reflexology	9 dropouts	Serum progesterone	None reported	Ovulation occurred in intervention (42%) and sham groups (46%), Pregnancy occurred in intervention (15%) and sham groups (9%), no sig. difference between groups	The authors stated that the rate of ovulation in this trial was double that expected giving rise to an idea that the sham treatment may also have had an effect on the outcome measures

Mackereth et al., 2009 [37] (UK) Crossover RCT with 4-week washout	53 MS patients	6 × 40 mins weekly reflexology (Ingham Method)	Progressive muscle relaxation (PRM) training	3 dropouts	HR and BP, salivary cortisol	None reported	Sig. decrease before and after treatment and before and after weeks 1–6 for cortisol within reflexology group, sig. decrease in SBP and HR for both groups, no sig. change in DBP, no sig. difference between groups	The variable of interest failed to return to initial levels resulting in problems relating to the ordering of the treatments and these interactions made analysis very difficult to determine

Hodgson and Andersen, 2008 [38] (USA) Crossover RCT with no washout period	21 dementia sufferers in nursing homes	4 × 30 mins weekly hand or foot reflexology	4 × 30 min weekly friendly visit	Not stated	BP, pulse, salivary *α*-amylase	None reported	Sig. decrease in salivary *α*-amylase in reflexology group, no other sig. results, significant between groups difference in amylase	The authors did not consider the impact of the absence of a washout period on results. Also, no details or numbers of patients receiving hand or foot reflexology were given

Gunnarsdottir and Jonsdottir, 2007 [39] (Iceland) Pilot RCT	9 Coronary artery bypass graft patients	5 × 30 mins reflexology (Ingham Method) pre and post-surgery	Cream application to feet (1 min) + 30 mins rest	2 due to post surgery complication	BP, HR, respiration rate	None reported	Sig reduction in SBP in control group, no sig. difference between groups	Anxiety levels in the control group were consistently lower in the control group and authors attribute higher anxiety scores to a potential lack of validity of SAI to the Icelandic population

Mc Vicar et al., 2007 [40] (UK) Pilot crossover RCT with 3-day washout	30 healthy volunteers	3 × 60 mins pragmatic reflexology	Sitting as a group in a quiet room	Not stated	Salivary melatonin & cortisol, BP, pulse rate	None reported	Significant reduction in pulse and SBD in reflexology group, no sig. change in DBP, no sig. change in cortisol or melatonin, no sig. difference between groups	Authors stated that carry over effects and order of treatments due to study design may have affected results. They also, suggest that sitting in a room as a group may have resulted in anxiety for some control participants

Wilkinson et al., 2006 [41] (UK) RCT	20 Chronic Obstructive Pulmonary Disease (COPD) patients	4 × 50 min sessions	Friendly visits	19 participants did not complete all of the study	BP, HR, respiration rate, oxygen saturation, FVC, FEV, vital capacity, peak flow	None reported	Significant pre-postdecrease in HR within reflexology group, no sig. improvement in HR and PEF in control group, no sig, difference between groups	Peak flows were self-reported and as only one participant completed all of the study, these results are open to bias

Mollart, 2003 [10] (Australia) Single-blind RCT	69 Pregnant women 30 weeks + gestation with foot oedema	3 × 15 mins session lymphatic reflexology	(1) Relaxing reflexology (2) Rest	Only 20 participants completed all 3 sessions	BP, ankle, and foot circumference measurements	None reported	Nonsignificant reduction in BP for all groups, nonsignificant decreases in ankle and foot measurements	Results from the first treatment session only were analysed due to dropouts

Frankel, 1997 [42] (UK) Pilot RCT	24 healthy participants	1 × 45 mins reflexology (Ingham Method)	(1) Foot massage (2) No intervention	None reported	Baroreceptor reflex sensitivity (BRS), BP, sinus arrhythmia (SA)	None reported	Nonsignificant reduction in BRS for reflexology and FM (60%) compared with no treatment (50%), nonsignificant increase in SA frequency for reflexology and FM, nonsignificant difference between groups	Author suggests a “Neuro theory” may explain the mechanism of action as BRS is under ANS control

ACC: anterior cingulate cortex; ANS: autonomic nervous system; BarDwEv: baroreceptor down events; BarUpEv: baroreceptor up events; BOLD: blood oxygen level dependent; BP: blood pressure; BRS: baroreceptor reflex sensitivity; CABG: coronary artery bypass graft; CAD: coronary artery disease; CHF: chronic heart failure; CI: cardiac index; CO: cardiac output; COPD: chronic obstructive pulmonary disease; DBP: diastolic blood pressure; ECG: electrocardiogram; FEV: forced expiration volume; FM: foot massage; fMRI: functional magnetic resonance imaging; FVC: forced vital capacity; HR: heart rate; HRV: heart rate variability; IBI: interbeat interval; PCC: posterior cingulate cortex; PEF: peak expiratory flow; PMR: progressive muscle relaxation; PP: pulse pressure; RCT: randomised controlled trial; RRI: R-R interval; SA: sinus arrhythmia; SAI: Spielbergers State Anxiety Inventory; SBP: systolic blood pressure; SI: Stroke Index; SIS: self-initiated support; TPR: total peripheral resistance.

**Table 5:** Assessment of quality using the GRADE system.

Number of studies and participants	Study limitations	Consistency of results	Directness of the evidence	Precision	Reporting bias	Overall quality of the evidence
17 RCTs and pilot studies (697 participants) start score = 4	−2 serious limitations due to problems with blinding	−2 serious inconsistency in results between studies	−1 some indirectness as most studies not comparable	−1 some imprecision due to low participant numbers	Unlikely as positive and negative effects found	Very low

## References

[1] House of lords select committee on science and technology sixth report (2000). http://www.publications.parliament.uk/pa/ld199900/ldselect/ldsctech/123/12301.htm.

[2] Lee J, Han M, Chung Y, Kim J, Choi J (2011). Effects of foot reflexology on fatigue, sleep and pain: a systematic review and meta-analysis. *Journal of Korean Academy of Nursing*.

[3] Jones J, Thomson P, Lauder W, Howie K, Leslie SJ (2012). Reflexology has an acute (immediate) haemodynamic effect in healthy volunteers: a double-blind randomised controlled trial. *Complementary Therapies in Clinical Practice*.

[4] McDonough S, Devine P, Baxter D Research update: complementary and alternative medicine: patterns of use in Northern Ireland. http://www.ark.ac.uk/publications/updates/update50.pdf.

[5] Ventola CL (2010). Current issues regarding complementary and alternative medicine (CAM) in the United States—part 1: the widespread use of CAM and the need for better-informed health care professionals to provide patient counseling. *Pharmacy and Therapeutics*.

[6] NIFAB-undersøkelsen 2007 http://www.nifab.no/om_alternativ_behandling/tall_og_fakta/nifab_undersoekelsen_2007.

[7] Danish Ministry of Health, Knowledge and Research Center for Alternative Medicine http://www.srab.dk/uk/alternative+medicine/reflexology.

[8] British Library http://www.bl.uk/bipc/aboutus/news/comptherapy.html.

[9] Hodgson H (2000). Does reflexology impact on cancer patients’ quality of life?. *Nursing Standard*.

[10] Mollart L (2003). Single-blind trial addressing the differential effects of two reflexology techniques versus rest, on ankle and foot oedema in late pregnancy. *Complementary Therapies in Nursing and Midwifery*.

[11] Stephenson NLN, Dalton JA (2003). Using reflexology for pain management. A review. *Journal of Holistic Nursing*.

[12] Wyatt G, Sikorskii A, Rahbar MA, Victorson D, You M (2012). Health-related quality-of-life outcomes: a reflexology trial with patients with advanced stage breast cancer. *Oncology Nursing Forum*.

[13] Botting D (1997). Review of literature on the effectiveness of reflexology. *Complementary Therapies in Nursing & Midwifery*.

[14] Ernst E, Köder K (1997). An overview of reflexology. *European Journal of General Practice*.

[15] Wang M-Y, Tsai P-S, Lee P-H, Chang W-Y, Yang C-M (2008). The efficacy of reflexology: systematic review. *Journal of Advanced Nursing*.

[16] Ernst E (2009). Is reflexology an effective intervention? A systematic review of randomised controlled trials. *Medical Journal of Australia*.

[17] Ernst E, Posadzki P, Lee MS (2011). Reflexology: an update of a systematic review of randomised clinical trials. *Maturitas*.

[18] Jones J, Thomson P, Irvine K, Leslie SJ (2013). Is there a specific haemodynamic effect in reflexology? A systematic review of randomised controlled trials. *The Journal of Alternative and Complementary Medicine*.

[19] Kim JI, Lee MS, Kang JW, Choi DY, Ernst E (2010). Reflexology for the symptomatic treatment of breast cancer: a systematic review. *Integrative Cancer Therapies*.

[20] Tiran D, Chummun H (2005). The physiological basis of reflexology and its use as a potential diagnostic tool. *Complementary Therapies in Clinical Practice*.

[21] Ingham E (1984). *Stories the Feet Can Tell Thru Reflexology, Stories the Feet Have Told Thru Reflexology*.

[22] Sudmeier I, Bodner G, Egger I, Mur E, Ulmer H, Herold M (1999). Changes of renal blood flow during organ-associated foot reflexology measured by colour doppler sonography. *Forschende Komplementarmedizin*.

[23] Dalal K, Elanchezhiyan D, Das R (2013). Noninvasive characterisation of foot reflexology areas by swept source-optical coherence tomography in patients with low back pain. *Evidence-Based Complementary and Alternative Medicine*.

[24] Universal College of Reflexology http://www.universalreflex.com/article.php/20040309175204417.

[25] Hughes CM, Krirsnakriengkrai S, Kumar S, McDonough SM (2011). The effect of reflexology on the autonomic nervous system in healthy adults: a feasibility study. *Alternative Therapies in Health and Medicine*.

[26] Sliz D, Smith A, Wiebking C, Northoff G, Hayley S (2012). Neural correlates of a single-session massage treatment. *Brain Imaging and Behavior*.

[27] Poole H, Glenn S, Murphy P (2007). A randomised controlled study of reflexology for the management of chronic low back pain. *European Journal of Pain*.

[28] Tiran D (2010). *Reflexology in Pregnancy and Childbirth*.

[29] Guyatt HG, Oxman AD, Vist GE (2008). GRADE: an emerging consensus on rating quality of the evidence and strength of recommendations. *British Medical Journal*.

[30] Jones J, Thomson P, Lauder W, Howie K, Leslie SJ (2013). Reflexology has no immediate haemodynamic effect in patients with chronic heart failure: a double-blind randomised controlled trial. *Complementary Therapies in Clinical Practice*.

[31] Hodgson NA, Lafferty D (2012). Reflexology versus Swedish massage to reduce physiologic stress and pain and improve mood in nursing home residents with cancer: a pilot trial. *Evidence-Based Complementary and Alternative Medicine*.

[32] Ruiz-Padial E, Torres-Lopez N, Luna-Bujaldón J, Espadas-Villanueva I, del Paso GR (2012). Cardiovascular effects of reflexology in healthy individuals: evidence for a specific increase in blood pressure. *Alternative Medicine Studies*.

[33] Lu WA, Chen GY, Kuo CD (2011). Foot reflexology can increase vagal modulation, decrease sympathetic modulation, and lower blood pressure in healthy subjects and patients with coronary artery disease. *Alternative Therapies in Health and Medicine*.

[34] Moeini M, Kahangi LS, Valiani M, Heshmat R (2011). The effect of reflexotherapy on patients' vital signs before coronary artery bypass graft surgery. *Iranian Journal of Nursing and Midwifery Research*.

[35] Green VL, Alexandropoulou A, Walker MB (2010). Alterations in the Th1/Th2 balance in breast cancer patients using reflexology and scalp massage. *Experimental and Therapeutic Medicine*.

[36] Holt J, Lord J, Acharya U (2009). The effectiveness of foot reflexology in inducing ovulation: a sham-controlled randomized trial. *Fertility and Sterility*.

[37] Mackereth PA, Booth K, Hillier VF, Caress AL (2009). Reflexology and progressive muscle relaxation training for people with multiple sclerosis: a crossover trial. *Complementary Therapies in Clinical Practice*.

[38] Hodgson NA, Andersen S (2008). The clinical efficacy of reflexology in nursing home residents with dementia. *Journal of Alternative and Complementary Medicine*.

[39] Gunnarsdottir TJ, Jonsdottir H (2007). Does the experimental design capture the effects of complementary therapy? A study using reflexology for patients undergoing coronary artery bypass graft surgery. *Journal of Clinical Nursing*.

[40] Mc Vicar AJ, Greenwood CR, Fewell F, D’arcy V, Chandrasekharan S, Alldridge LC (2007). Evaluation of anxiety, salivary cortisol and melatonin secretion following reflexology treatment: a pilot study in healthy individuals. *Complementary Therapies in Clinical Practice*.

[41] Wilkinson ISA, Prigmore S, Rayner CF (2006). A randomised-controlled trail examining the effects of reflexology of patients with chronic obstructive pulmonary disease (COPD). *Complementary Therapies in Clinical Practice*.

[42] Frankel BSM (1997). The effect of reflexology on baroreceptor reflex sensitivity, blood pressure and sinus arrhythmia. *Complementary Therapies in Medicine*.

[43] Moher D, Liberati A, Tetzlaff J, Altman DG (2009). Preferred reporting for systematic reviews and meta-analyses. *PLoS Medicine*.

[44] Higgins JPT, Green S (2011). *Cochrane Handbook for Systematic Reviews of Interventions Version 5.1.0*.

[45] Brygge T, Heinig JH, Collins P (2001). Reflexology and bronchial asthma. *Respiratory Medicine*.

[46] Bradt J, Dileo C (2009). Music for stress and anxiety reduction in coronary heart disease patients. *Cochrane Database of Systematic Reviews*.

[47] Marquardt H (2007). *Reflex Zone Therapy of the Feet: A Comprehensive Guide for Health Professionals*.

[48] Tiran D, Mackereth PA (2011). *Clinical Reflexology: A Guide for Integrated Practice*.

[49] National Institute for Health and Care Excellence (NICE) Clinical guidelines hypertension: clinical management of primary hypertension in adults. http://publications.nice.org.uk/hypertension-cg127.

